# *Chlamydiae* Assemble a Pathogen Synapse to Hijack the Host Endoplasmic Reticulum

**DOI:** 10.1111/tra.12002

**Published:** 2012-09-11

**Authors:** Maud Dumoux, Daniel K Clare, Helen R Saibil, Richard D Hayward

**Affiliations:** 1 Institute of Structural and Molecular Biology, Birkbeck & University College LondonMalet Street, London, WC1E 7HX, UK; 2 Department of Crystallography, Institute of Structural and Molecular BiologyBirkbeck, Malet Street, London, WC1E 7HX, UK

**Keywords:** *Chlamydia*, endoplasmic reticulum, microscopy, pathogenesis, type III secretion

## Abstract

*Chlamydiae* are obligate intracellular bacterial pathogens that replicate within a specialized membrane-bound compartment, termed an ‘inclusion’. The inclusion membrane is a critical host–pathogen interface, yet the extent of its interaction with cellular organelles and the origin of this membrane remain poorly defined. Here we show that the host endoplasmic reticulum (ER) is specifically recruited to the inclusion, and that key rough ER (rER) proteins are enriched on and translocated into the inclusion. rER recruitment is a *Chlamydia*-orchestrated process that occurs independently of host trafficking. Generation of infectious progeny requires an intact ER, since ER vacuolation early during infection stalls inclusion development, whereas disruption post ER recruitment bursts the inclusion. Electron tomography and immunolabelling of *Chlamydia*-infected cells reveal ‘pathogen synapses’ at which ordered arrays of chlamydial type III secretion complexes connect to the inclusion membrane only at rER contact sites. Our data show a supramolecular assembly involved in pathogen hijack of a key host organelle.

*Chlamydiae* are obligate intracellular bacterial pathogens that remain a significant cause of sexually transmitted infections worldwide, and preventable blindness in developing nations. Infectious chlamydial elementary bodies (EBs) force their own entry into mammalian epithelial cells by triggering actin-dependent membrane ruffling. Once internalized, EBs differentiate into larger, reticulate bodies (RBs), which replicate within a specialized membrane-bound compartment termed an inclusion. Following subsequent RB–EB reversion, bacterial progeny exit to infect neighbouring cells (1).

*Chlamydia* entry, bacterial replication and inclusion biogenesis are controlled by bacterial virulence effector proteins, which are delivered into the inclusion lumen, the inclusion membrane and the host cell nucleus and cytosol. The delivery of many of these effectors depends on a type III secretion system (T3SS), which is similar to those present in other gram-negative bacteria (1–4). Indeed, macromolecular complexes have been observed spanning chlamydial membranes (5–7), and although these have been proposed to represent T3SSs (8), their precise identity remains a subject of debate (9).

Although most of the underlying effector mechanisms are unknown, it is clear that the inclusion remains segregated from the host endocytic pathway. Nevertheless, despite remaining impermeable to small molecules (10), the inclusion selectively intercepts vesicles from the secretory pathway to acquire cholesterol and sphingolipids (11,12), in part by effector-mediated hijack of cellular transporters that transiently interact with the Golgi and endoplasmic reticulum (ER) (13,14). Remarkably, intact lipid droplets are also engulfed from the host cytosol (15). The inclusion membrane is thus a critical intracellular interface for host–pathogen communication, yet its transport processes and the extent of its interaction with cellular organelles remain poorly defined. In this study, we investigated interactions between the *Chlamydia trachomatis* inclusion and the host rough endoplasmic reticulum (rER). We reveal a novel structure and termed it a ‘pathogen synapse’ interconnecting the bacterial T3SS, the inclusion membrane and the host rER.

## Results

### Mature chlamydial inclusions engage the host ER

**Figure 1:**
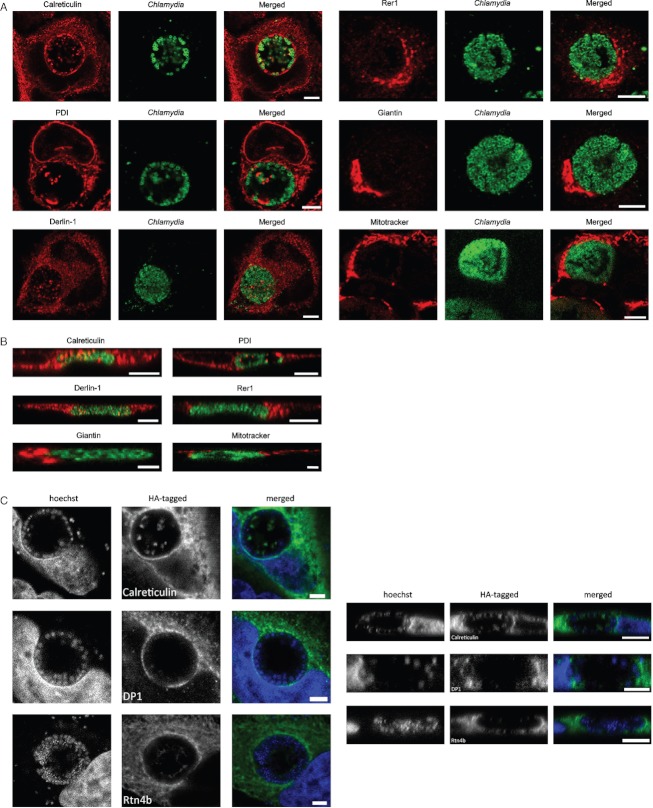
The *Chlamydia* inclusion specifically recruits ER markers A) Confocal xy-sections of HeLa cells after infection with *C. trachomatis* LGV2 and fixed 24 hpi. Cells were immunolabelled for ER, intermediate compartment or Golgi markers, or incubated with Mitotracker Orange prior to fixation as indicated (red), and co-stained for *Chlamydia* (green). Scale bars, 5 µm. B) Confocal xz sections as (A), acquired with a y-step of 5 µm. Scale bars, 5 µm. C) Confocal xy-sections of HeLa HA-calreticulin, DP1-HA and Rtn4b-HA transfectants fixed 24 hpi with *C. trachomatis* LGV2. Cells were immunolabelled for HA (green) and stained with Hoechst. Right panels show corresponding xz-stacks acquired with a y-step of 5 µm. Scale bars, 5 µm.

To probe possible interactions between bacterial inclusions and the host ER, HeLa cells were initially fixed 24 hours post-infection (hpi) with *Chlamydia trachomatis* LGV2, and mid-stage inclusions containing replicative chlamydial RBs (1) examined in relation to the ER marker calreticulin (16) by confocal microscopy. Calreticulin was significantly enriched at the inclusion periphery and accumulated in patches at the inclusion membrane proximal to luminal RBs. Calreticulin was also detected within the inclusion itself, often in close contact with the bacteria (Figure 1A,B, calreticulin). Examination of serial confocal z-sections confirmed that calreticulin accumulation occurred over the entire inclusion surface (Figure S1). To verify whether the calreticulin signal observed within the inclusion in the confocal xy-plane was truly luminal (Figure 1B, calreticulin), the locations of calreticulin and a cytoplasmic protein, F-actin, were compared in confocal xz-sections. F-actin only accumulates at the inclusion periphery (17), and there was no significant colocalization between F-actin and bacteria. By comparison, calreticulin was frequently localized adjacent to bacteria in the inclusion lumen (Figure S2). To further verify these observations, HeLa cells expressing a calreticulin derivative containing an *N*-terminal hemagglutinin epitope tag (HA-calreticulin) were infected with *C. trachomatis* LGV2 and examined analogously 24 hpi following immunostaining with an anti-HA monoclonal antibody. Even in the presence of endogenous calreticulin, HA-calreticulin was also enriched at the inclusion periphery and translocated into the inclusion lumen (Figure 1C, HA-calreticulin).

To investigate whether the distribution of other archetypal ER marker proteins mirrored that of calreticulin, we next examined the localization of protein disulphide isomerase (PDI) in *Chlamydia*-infected cells (18). Like calreticulin, PDI was also strikingly enriched in patches at the inclusion periphery and associated with bacteria inside the inclusion (Figure 1A,B, PDI). Der1-like protein 1 (derlin-1), a transmembrane ER protein involved in ER-associated degradation (ERAD; (19) also entered the inclusion, but in contrast to calreticulin and PDI did not accumulate significantly at the inclusion membrane. The comparatively low levels of derlin-1 expression observed imply that generic ER stress is not induced in the infected cells (Figures 1A & 1B, Derlin-1). In contrast, retrieval to ER protein 1 (Rer1), a protein that dynamically associates with intermediate compartments and the *cis*-Golgi (20), and giantin a *cis*- and medial-Golgi tethrin (21) were never observed enriched at the inclusion periphery or present in the inclusion (Figures 1A & 1B,; Rer1, giantin). Similarly, live mitochondria never specifically accumulated at the inclusion (Figure 1A, mitotracker) when analysed under the same conditions. To establish whether ER scaffold proteins are recruited to the mature inclusion, HeLa cells expressing epitope-tagged reticulon Rtn4b and its interacting protein deleted in polyposis (DP-1; (22) were infected with *C. trachomatis* LGV2. Both Rtn4b-HA and DP-1-HA were also clearly enriched on the inclusion at late stages of the infection cycle (Figure 1C).

**Figure 2:**
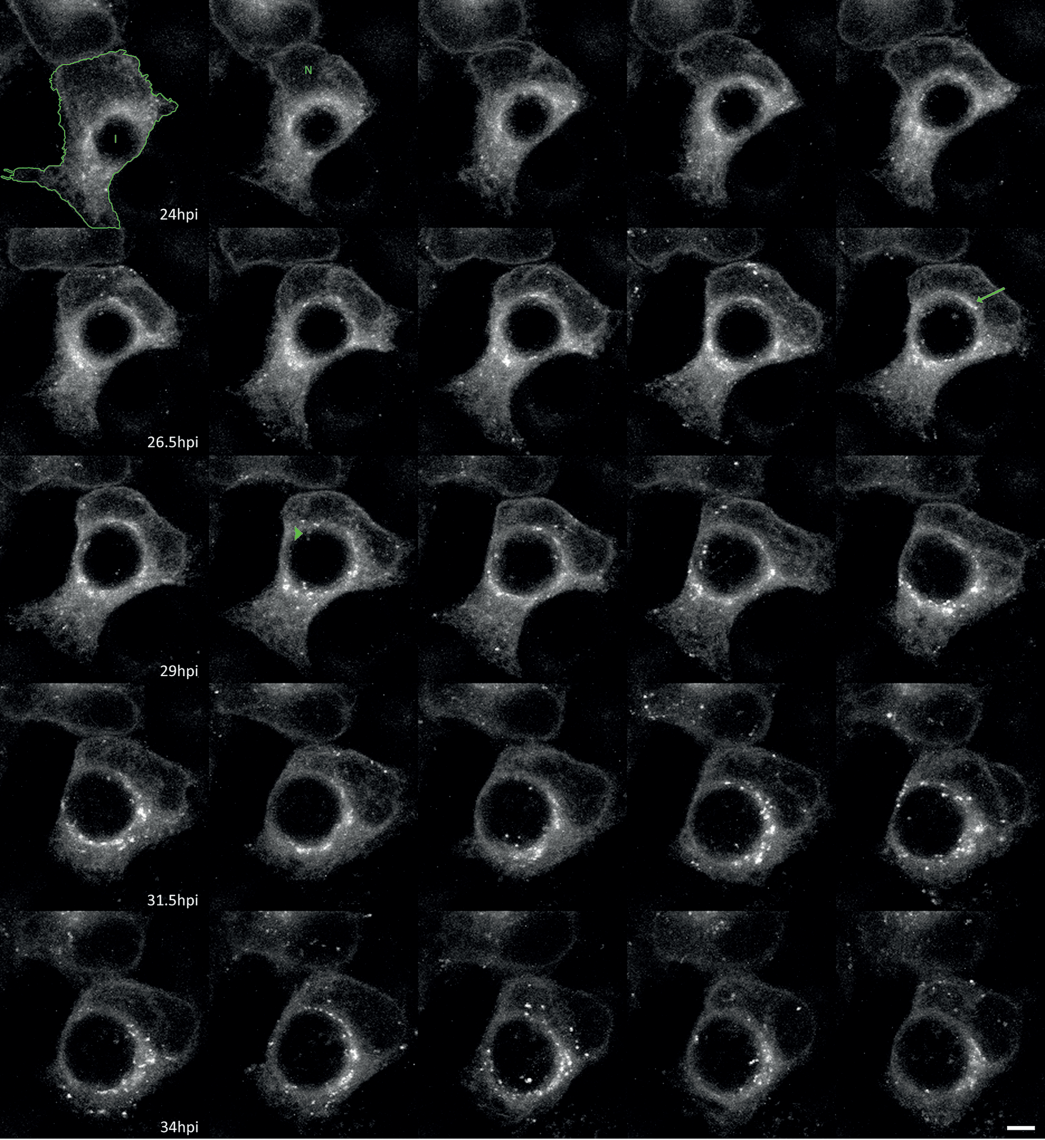
DsRed-ER is recruited to the chlamydial inclusion in live cells HeLa DsRed-ER transfectants were infected with *C. trachomatis* LGV2 and individual cells imaged by confocal microscopy beginning 24 hpi. Panels show maximum intensity projections of confocal stacks of a typical infected cell imaged every 30 min. Cell periphery (outline), inclusion (I) and cell nucleus (N) are indicated in the first two panels for clarity. DsRed-ER (greyscale) accumulates at the inclusion periphery (an example indicated with an arrow in panel 10), and translocates into the inclusion lumen (an example indicated with an arrowhead in panel 12). Scale bar, 5 µm.

To verify these results obtained from infected cells after fixation, cells expressing Discosoma red fluorescent protein (DsRed) fused to both the ER-targeting sequence of calreticulin and the ER retention signal KDEL (DsRed-ER) were infected with *C. trachomatis* LGV2 and imaged from 24 hpi using live confocal microscopy. DsRed-ER accumulated in patches at the inclusion periphery and was translocated into the inclusion lumen (Figure 2, Movie S1), a phenotype identical to that observed in the fixed samples. We additionally exploited a highly selective and photostable ER-Tracker Blue-White DPX live cell probe (Invitrogen) to examine ER dynamics using fluorescence video microscopy. The probe partitioned rapidly and specifically into the ER upon addition to cultured cells, and in infected cells clearly labelled inclusions as well as the ER (Figure S3A, Movie S2). Quantification of the relative fluorescence signals revealed a fast initial rate of probe uptake into the inclusions, which then remained labelled at a constant level, at a lower intensity than the neighbouring ER for the duration of the experiment (Figure S3B). These observations show that an ER-targeted reporter protein and an ER-specific live probe label mature inclusions in agreement with our data from the fixed cells, and suggest that these inclusions potentially adopt sufficient ER-like character to permit labelling.

**Figure 3:**
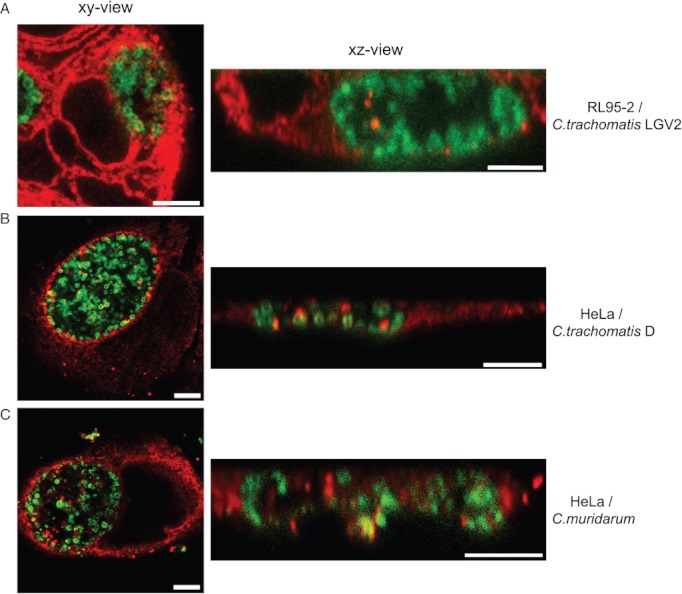
Calreticulin recruitment is independent of cell type and *Chlamydia* species/serovar For each cell type and *Chlamydia* strain, fixation was performed at 24 hpi followed by immunolabelling for calreticulin (red) and *Chlamydia* (green). xy- and xz-stacks were acquired by confocal microscopy. Scale bars, 5 µm. A) Endometrial cell line RL95-2 infected with *C. trachomatis* LGV2. B) Cervical cell line HeLa infected with *C. trachomatis* serovar D. C) HeLa cells infected with *Chlamydia muridarum*.

Using endogenous calreticulin as a representative ER marker, we next confirmed that these observations were independent of host cell type and *Chlamydia* species or serovar, as analogous localization of calreticulin was evident when HeLa cells were infected with *C. trachomatis* serovar D, related *Chlamydia muridarum* or when human endometrial cells were infected with *C. trachomatis* LGV2 (Figure 3).

Together, our findings reveal a surprisingly extensive and specific interaction between mature chlamydial inclusions and luminal (18), transmembrane (19) and scaffolding proteins (22) characteristic of the ER, which are enriched on and within the inclusion.

### Biphasic recruitment of the host ER during the chlamydial infection cycle

**Figure 4:**
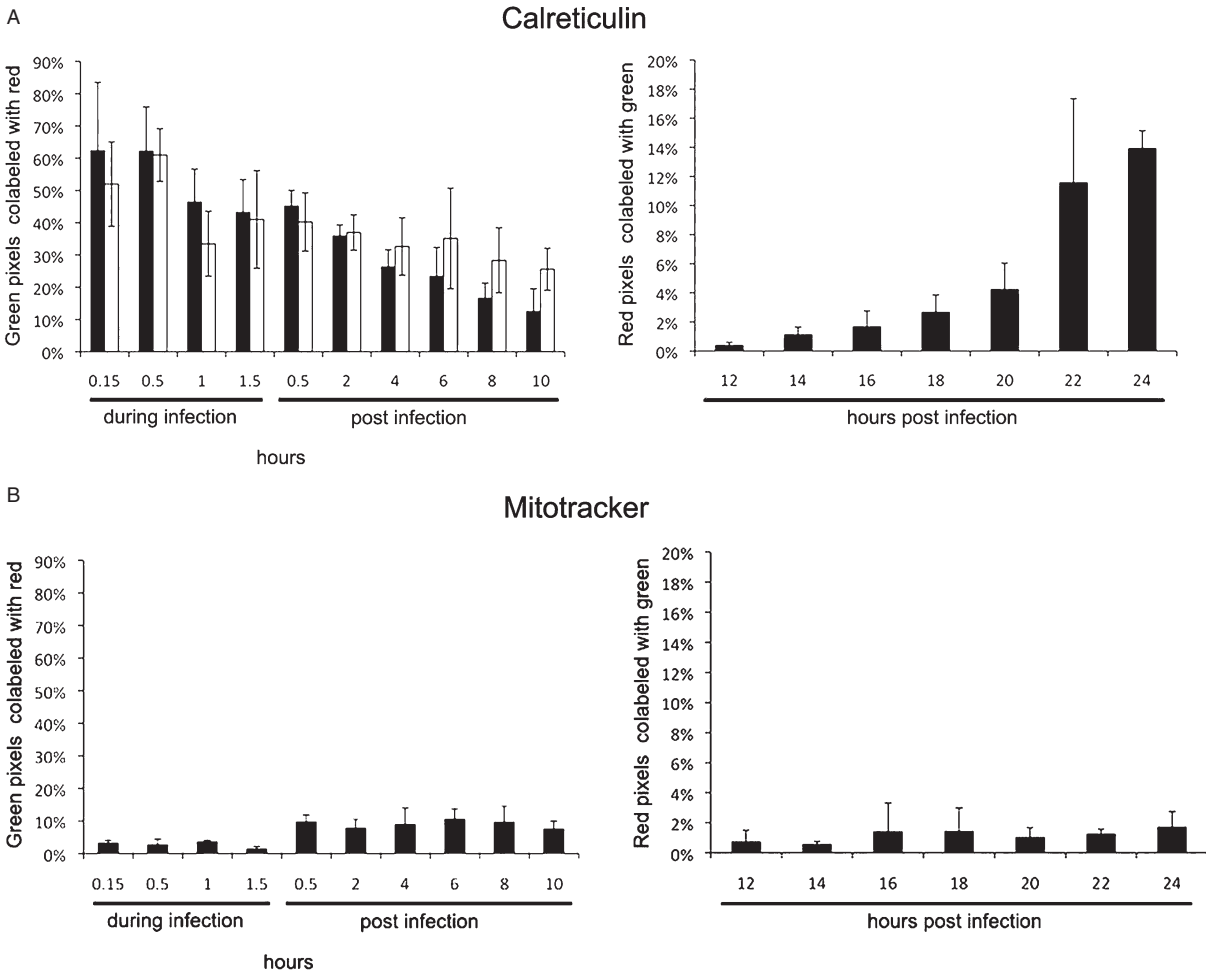
Biphasic recruitment of ER during the *Chlamydia* infection cycle A) Quantitative analysis of calreticulin recruitment during the *Chlamydia* infection cycle. HeLa cells were infected with *C. trachomatis* LGV2 and fixed at different times post-infection. Left plot shows early time points when bacteria are not yet organized as an inclusion. The behaviour of live (black) and heat-killed (white) bacteria is not statistically different (p < 0.05). Right plot shows later stages, when bacteria are progressively organized into a mature inclusion. B) Quantitative analysis of Mitotracker Orange recruitment during the *Chlamydia* infection cycle. Left plot shows early time points when bacteria are not yet organized as an inclusion. Right plot shows later stages, when bacteria are progressively organized into a mature inclusion.

**Figure 5:**
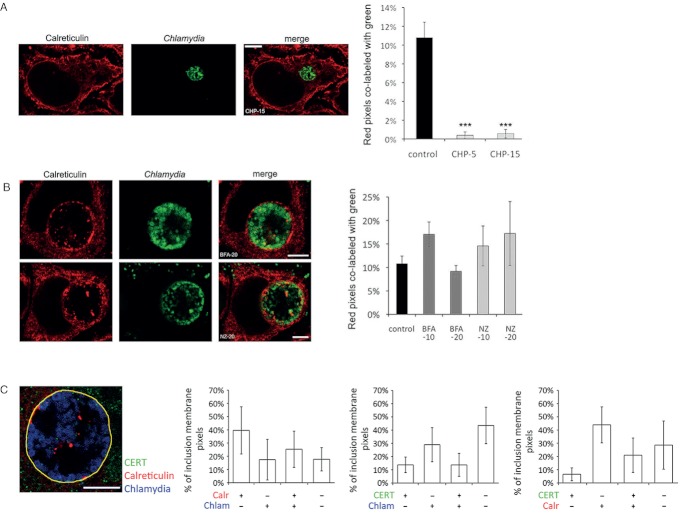
Calreticulin recruitment requires bacterial protein synthesis, but occurs independently of ER-Golgi trafficking A) HeLa cells were infected with *C. trachomatis* LGV2, treated with chloramphenicol 12 hpi (5 or 15 µg/mL; CHP-5, CHP-15), and fixed 24 hpi. Calreticulin (red) and *Chlamydia* (green) were immunolabelled and imaged by confocal microscopy. Scale bar, 5 µm. Quantification of calreticulin at the inclusion following chloramphenicol treatment was performed using the second Manders coefficient (red pixels colabelled green) from confocal z-sections. Error bars represent standard deviation (***p < 0.001). B) HeLa cells were infected with *C. trachomatis* LGV2. Brefeldin A (14 hpi; BFA, 10 or 20 µg/mL; BFA-10, BFA-20) or nocodazole (14 hpi; NZ, 10 or 20 ng/mL; NZ-10, NZ-20) was added to the media and cells fixed 24 hpi. Cells were immunolabelled for calreticulin (red) and *Chlamydia* (green) and z-stacks acquired by confocal microscopy. Upper panels show confocal xy-section of a typical infected cell treated with 20 µg/mL BFA or 20 ng/mL NZ. Scale bars, 5 µm. Quantification of calreticulin at the inclusion was performed using the second Manders coefficient (red pixels colabelled green) from z-sections. No significant differences were observed between the control and the treated cells (p < 0.05). Error bars represent the standard deviation. C) HeLa cells were fixed 24 hpi with *C. trachomatis* LGV2 and immunolabelled for CERT (green), calreticulin (red) and *Chlamydia* (AlexaFluor 633 pseudocoloured blue). Left panel shows a representative confocal xy-section through a chlamydial inclusion containing typical calreticulin and CERT patches. The contour of the inclusion used to analyse the grey levels in each channel is shown in yellow. Scale bar, 5 µm. Histograms show pairwise comparisons of each channel following analysis of multiple inclusions, illustrating the percentage of inclusion membrane pixels stained in each channel, alone or in combination as indicated.

Having established the general nature of our observations in live and fixed cells, we next quantified the extent of colocalization between bacteria-containing compartments and the ER during the entire chlamydial infection cycle. Manders colocalization coefficients 1993 were calculated at different time points, allowing an unbiased evaluation of the degree of overlap between the two fluorescence signals. The results show a biphasic interaction. Initially, calreticulin was recruited during cell entry and to early vacuoles containing chlamydial EBs (1), and was then progressively lost. Interaction increased dramatically in the later stages of the cycle when bacteria are organized in an inclusion (1), with 13.9 ± 1.2% of cellular calreticulin recruited (Figure 4A). An equivalent analysis showed no significant colocalization between active mitochondria and inclusions (Figure 4B). Whereas heat-killed and live bacteria behaved equivalently during the early phase (Figure 4A), inhibition of bacterial protein synthesis during the late phase abolished significant calreticulin recruitment (Figure 5A). These data show an early *Chlamydia*-independent interaction of calreticulin-positive membranes with bacteria-containing vesicles during and immediately after entry, perhaps in agreement with reported roles for the ER during phagocytosis (24); conversely, they show that later during infection *Chlamydia* actively recruits the same endomembranes to the mature inclusion at a precise time point.

### ER-inclusion interaction occurs independently of ER–Golgi trafficking

As some pathogens like *Legionella* and *Brucella* subvert ER–Golgi trafficking to indirectly recruit ER membranes to their replicative compartment (25), and *Chlamydia* harnesses sphingomyelin from the secretory pathway (26), we next probed host requirements for ER-inclusion interaction using calreticulin as a reporter. Treatment of infected cells with brefeldin A (BFA) or nocodazole (NZ), which block ER–Golgi and generic microtubule-dependent transport processes respectively, failed to prevent significant calreticulin accumulation around or within the inclusion (Figure 5B). These data show that in contrast to some *Chlamydia*-directed processes (13,26), calreticulin is not recruited into the inclusion via vesicle capture from ER–Golgi traffic.

Recently ceramide transfer protein (CERT), a cytosolic host protein, has been implicated in ceramide transport into the inclusion, possibly via the ER (13,14). As CERT interacts transiently with the Golgi and ER by binding phosphatidylinositol-4-phosphate and cellular VAT proteins respectively (27), we investigated whether there is any relationship between CERT and calreticulin recruitment to mature inclusions. Endogenous CERT was also enriched in patches on the inclusion membrane at 24 hpi, although to a lesser extent than calreticulin (Figure 5C). Analysis and rendering of triple-labelled confocal sections revealed that some regions of the inclusion membrane contained both CERT and calreticulin, which might be expected stochastically given their shared capacity for ER localization (Figure 5C). However, most patches proximal to bacteria were enriched in either CERT or calreticulin, calreticulin being observed more prevalently than CERT (Figure 5C). CERT also entered the inclusion, predominantly as small luminal puncta, and was occasionally observed in larger vesicular structures. These luminal CERT-containing structures were distinct from the calreticulin associated with the bacterial surface (Figure S4; Movies S3 and S4). These data show that a minor fraction of calreticulin-positive regions of the inclusion membrane also contain CERT and therefore may contribute to the previously identified ceramide transport pathway (13,14). However, distinct calreticulin-containing structures are more frequently observed at the inclusion membrane, suggesting multiple roles for ER-derived structures in inclusion biogenesis.

### Generation of infectious bacterial progeny requires an intact ER

**Figure 6:**
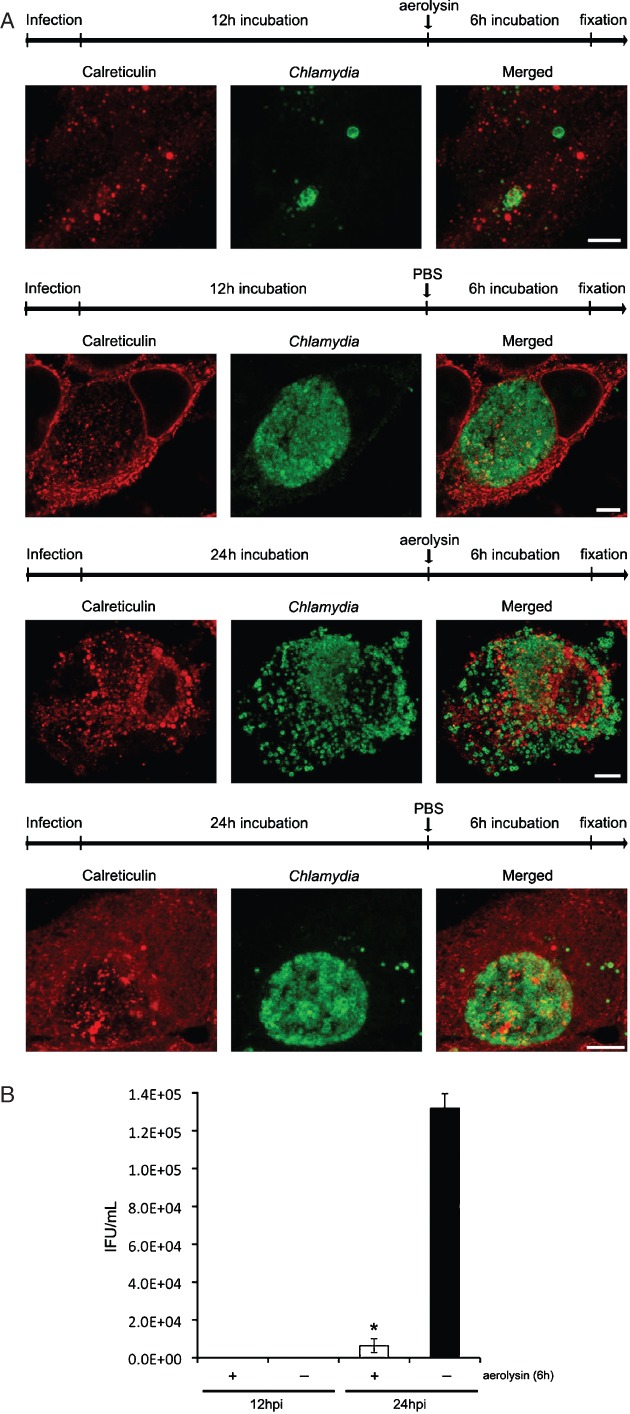
Aerolysin treatment disturbs inclusion biogenesis and abolishes infectivity A) HeLa cells were infected with *C. trachomatis* LGV2. At 12 or 24 hpi, cells were treated (30 min) with pro-aerolysin (0.5 nm) and fixed 6 h later for immunolabelling for calreticulin (red) and *Chlamydia* (green). When aerolysin is added 12 hpi (upper), vacuolization inhibits growth of the inclusion and prevents calreticulin recruitment. When added 24 hpi (lower), the inclusion bursts and RBs disseminate into the cytosol. Scale bars, 5 µm. B) Cell layers collected after infection and treatment with aerolysin (A) were diluted in fresh medium to infect a new layer of HeLa cells. After 24 h, freshly infected cells were fixed and stained for *Chlamydia* and DNA. Random fields were scored to quantify inclusion-forming units per mL (IFU/mL). Error bars represent standard deviation (*p < 0.05). It was not possible to assay the effect on bacterial infectivity 12 hpi as all the intracellular bacteria in the control and treated cells were in the non-infectious RB state.

**Figure 7:**
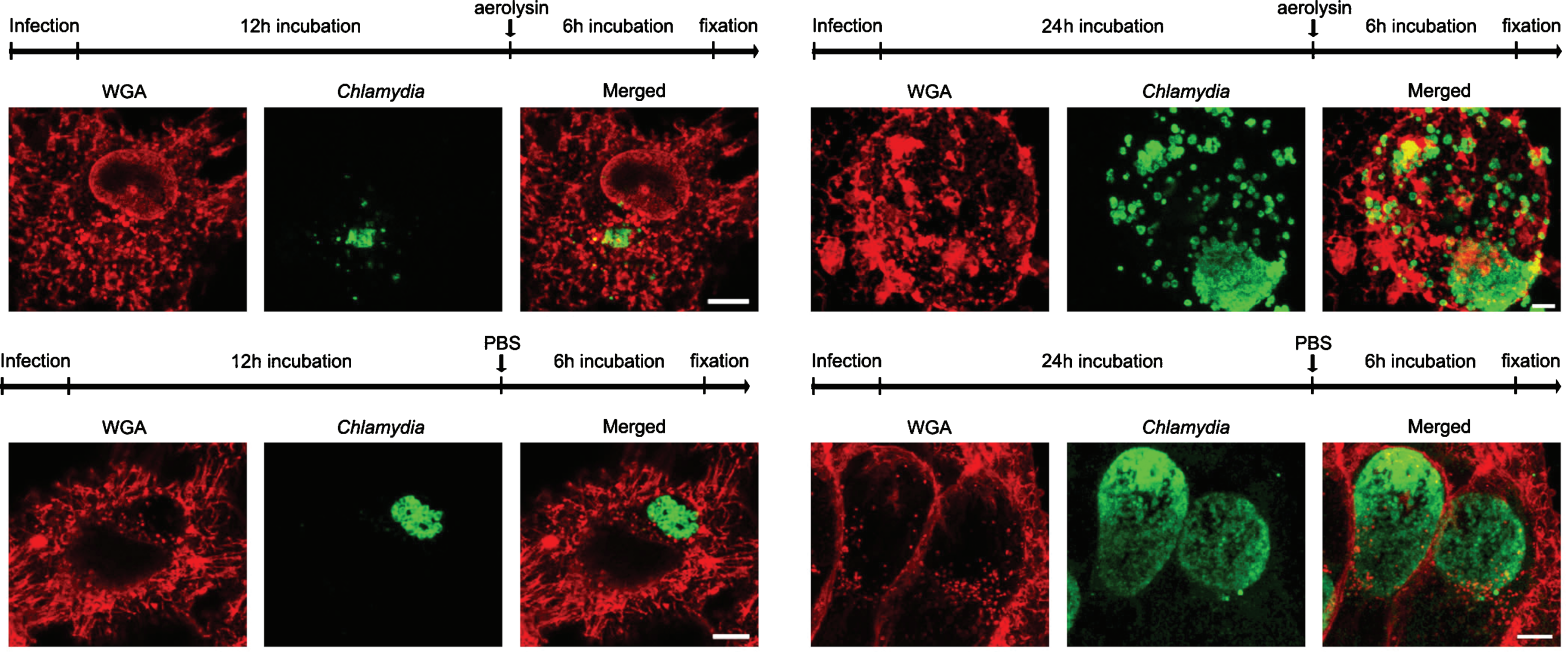
Aerolysin treatment arrests inclusion growth but maintains cell integrity HeLa cells were infected with *C. trachomatis* LGV2. At 12 or 24 hpi, cells were treated 30 min with 0.5 nm pro-aerolysin and fixed for immunolabelling 6 h later. AlexaFluor 594-coupled wheat germ agglutinin (WGA) (red) was incubated with cells 15 min prior to *Chlamydia* immunolabelling (green). WGA recognizes carbohydrates present predominantly at the plasma membrane and in early endocytic vesicles. Aerolysin treatment inhibits *Chlamydia* inclusion development when cells are treated 12 hpi. When aerolysin is added 24 hpi, once the ER is already recruited, the inclusion membrane is disrupted and bacteria released into the cell cytoplasm. Scale bars, 5 µm. Images are average projections of 4 xy-views in z to facilitate complete visualization of the plasma membrane, representing a thickness of 1.32 µm.

**Figure 8:**
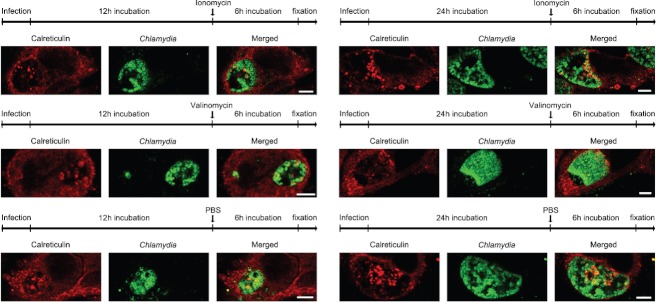
Ionophores do not affect *Chlamydia* growth or inclusion membrane integrity HeLa cells were infected with *C. trachomatis* LGV2. At 12 or 24 hpi, cells were treated 30 min with 2 μm of ionomycin or valinomycin, calcium and potassium ionophores, respectively. After 6 h, cells were fixed and immunolabelled for calreticulin (red) and *Chlamydia* (green). Scale bars, 5 µm.

To test whether ER recruitment is functionally important for inclusion biogenesis, we exploited the capacity of the aerolysin toxin from *Aeromonas hydrophila* to trigger specific vacuolation of the ER without disruption of the Golgi or endocytic compartments (28). *Chlamydia*-infected cells were treated with 0.5 nm purified pro-aerolysin 12 and 24 hpi followed by a further 6 h incubation. Calreticulin staining confirmed extensive vacuolation of the ER (Figure 6A). Strikingly, treatment at 12 hpi resulted in the arrest of early inclusion expansion, whereas addition of pro-aerolysin at 24 hpi destroyed the integrity of the mature inclusion, resulting in the dispersion of bacteria presumably into the cytosol of the cells, which remain intact as confirmed by membrane staining (Figures 6A and 7). These observations were due to disruption of the ER and not to toxin-induced flux of potassium or calcium ions (28), as treatment with ionophores did not induce any similar effects (Figure 8). Moreover, aerolysin-induced release of bacteria from mature inclusions was associated with a dramatic attenuation of infectivity (Figure 6B). These data show that bacterial hijack of the ER is required not only for inclusion biogenesis and inclusion membrane integrity, but is also linked to chlamydial growth and infectivity.

### Pathogen synapses: ordered connections between the chlamydial T3SS, inclusion membrane and host ER

**Figure 9:**
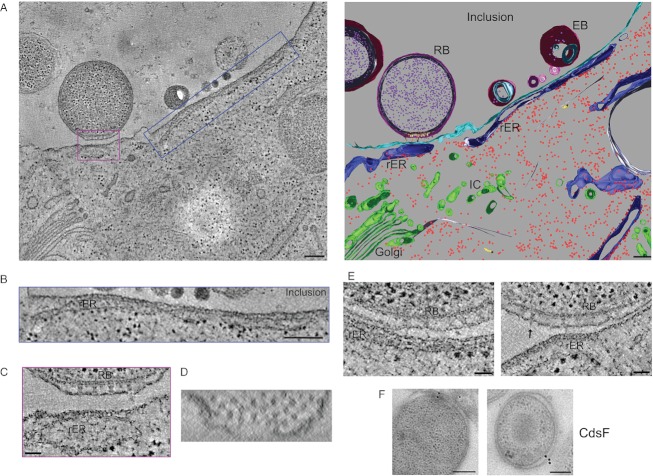
Electron tomography reveals a pathogen synapse involving the ER HeLa cells were infected with *C. trachomatis* LGV2 and high pressure frozen at 24 hpi. Tomograms were recorded from 200 nm thick sections of the freeze-substituted, embedded samples. A) Left panel shows an average of 10 z-sections after reconstruction, alignment and de-noising. The tomogram shows an example of an rER-inclusion contact. Manual segmentation (right panel) reveals the 3D organization. There is widespread contact between the inclusion membrane (bright blue) and rER (dark blue; eukaryotic ribosomes in red). RBs proximal to the inclusion membrane contain structures (brown) originating in the inner membrane (dark grey) and traversing the outer membrane (purple) to contact the inclusion membrane. Smaller, prokaryotic ribosomes are violet. Golgi and intermediate compartments (IC; green) do not contact the inclusion. Scale bars, 200 nm. B) Enlargement of the blue-boxed area in (A) showing an extensive rER-inclusion contact. Scale bar, 200 nm. (C) Enlargement of the purple-boxed area in (A) showing structures traversing the chlamydial membranes. Scale bar, 50 nm. D) Cross-section of the structures in (C) traversing the periplasm. E) Sections of two pathogen synapses showing needles in contact with the inclusion membrane–host ER contact sites and tip complexes (arrow). Scale bar, 50 nm. F) Immunogold CdsF labelling showing an RB involved in a pathogen synapse (left) and an EB (right). Scale bars, 250 nm (left) and 100 nm (right).

**Figure 10:**
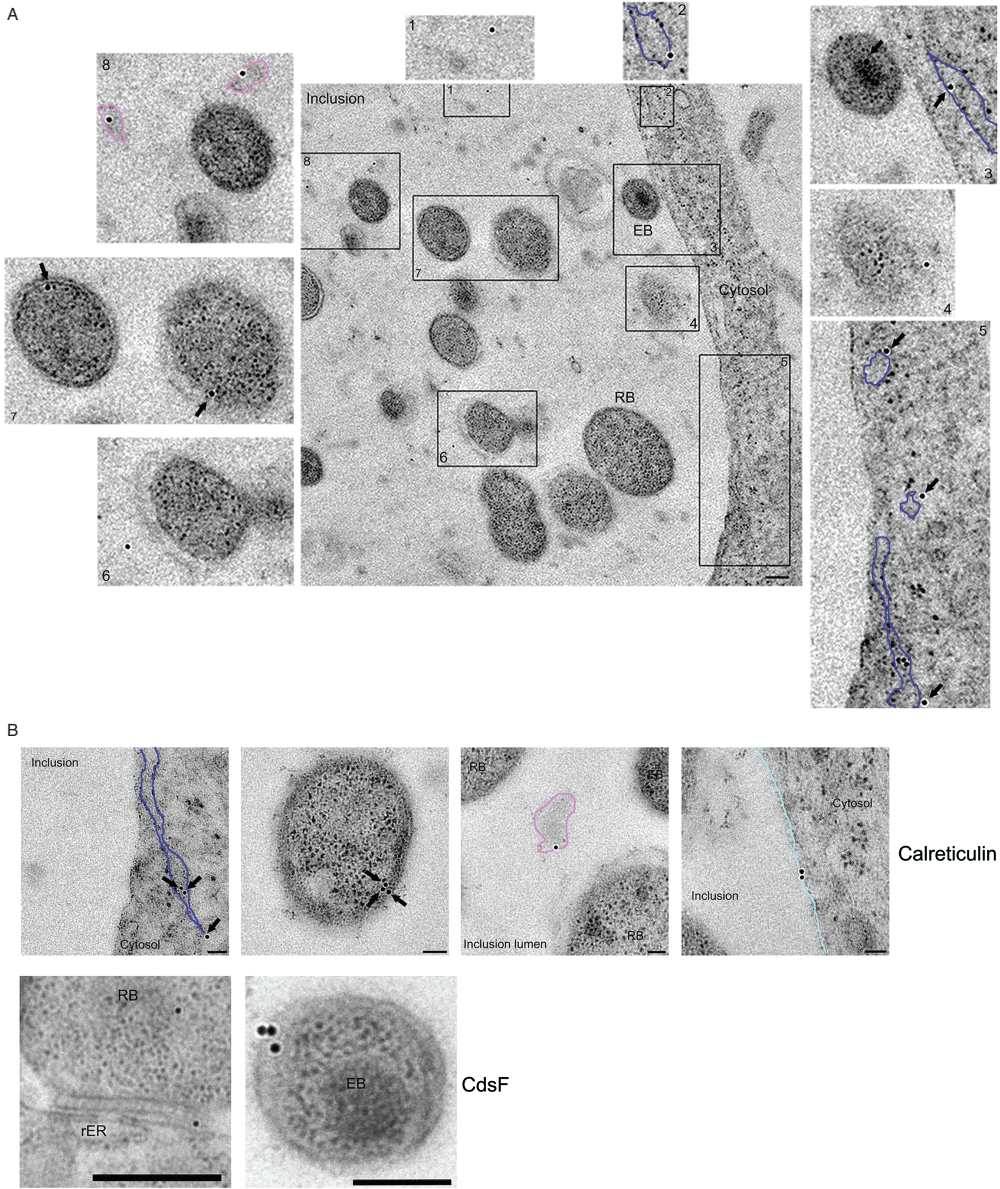
Immunogold labelling reveals a pathogen synapse with the inclusion membrane apposed to the rER A) Lower magnification overview of part of a *Chlamydia* inclusion showing immunogold labelling of calreticulin (10 nm). Labelling is specifically observed at the ER membrane (blue tracing; panels 2, 3 and 5), at the bacterial membrane (panels 4, 6 and 7) and on free membrane fragments in the inclusion lumen (pink tracing; 1, 6 and 8). Scale bar, 1 µm. B) 50-nm thickness sections were immunolabelled with anti-calreticulin or anti-CdsF primary and a gold-conjugated secondary antibody. Upper panels show typical anti-calreticulin immunogold labelling on rER membranes (dark blue) contacting the inclusion membrane, on the bacterial membrane, on free membrane fragments in the inclusion (outlined in pink) and on the inclusion membrane (light blue). Scale bars, 50 nm. Lower panels show additional examples of immunogold labelling of CdsF (10 nm) showing an RB involved in a ‘pathogen synapse’ (left) and an EB (right). CdsF is a major component of the T3SS. Scale bars, 500 nm (left) and 250 nm (right).

To investigate the interacting structures in more detail by electron microscopy, unfixed HeLa cells infected with *C. trachomatis* LGV2 were vitrified at 24 hpi by high pressure freezing (HPF), freeze-substituted and embedded in Lowicryl for EM sectioning. Tomograms recorded from these sections revealed frequent close apposition of ribosome-studded rough ER (rER) and the cytoplasmic face of the inclusion membrane. Contact between the rER and the inclusion was intimate and extensive, occurring for substantial distances along the inclusion membrane, with the host ribosomes partitioned onto the inclusion distal side of the rER tubules (Figures 9A, 9B and S5). Immunogold labelling confirmed that calreticulin was present on inclusion-apposed rER membranes, the inclusion membrane itself, on membrane fragments observed within the inclusion lumen and on the surface of bacteria (Figure 10A), in agreement with the confocal immunofluorescence data (Figures 1A and S1).

**Figure 11:**
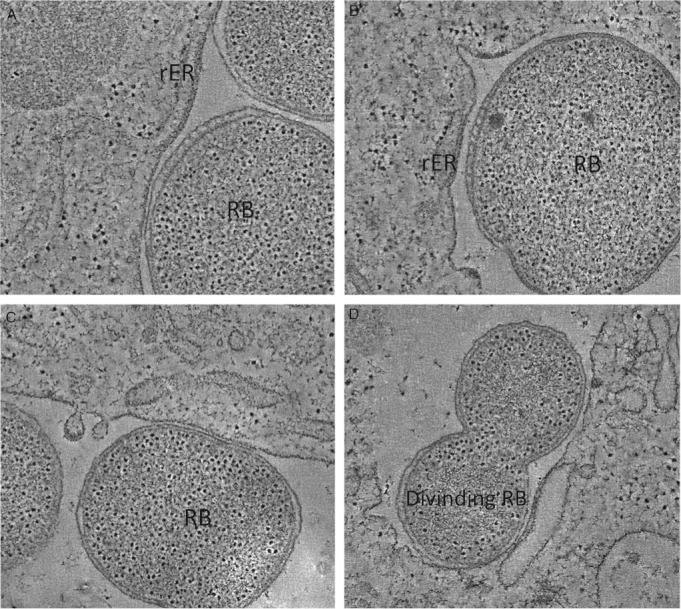
T3SS assemblies at rER-inclusion contact sites with luminal RBs HeLa cells were infected with *C. trachomatis* LGV2 and high pressure frozen at 24 hpi. Tomograms of 200 nm sections, showing RBs in contact with the inclusion membrane. T3SS complexes are observed when RBs contact an inclusion membrane connected to the rER (A and B), but not in the absence of ER-inclusion interaction (C) or when a smooth membrane contacts the inclusion (D). Scale bars, 100 nm.

The tomograms also revealed macromolecular complexes spanning both RB membranes in contact with the luminal face of the inclusion membrane at sites coincident with rER recruitment on the cytoplasmic face (Figure 9C, Movie S5). The dimensions of these complexes, which are 18 nm in diameter at the inner membrane with a 33 nm long rod spanning the periplasm, closely resemble those of isolated T3SS substructures from other gram-negative bacteria (29). These complexes assembled into localized arrays (Figure 9D), each containing 20–100 assemblies, which were associated with a pronounced widening of the periplasmic space (Figure 9C). Although only 5 nm in diameter, needle structures could also be detected extending from the bacterial surface, connecting the outer membrane and the inclusion membrane, or as isolated tip complexes (Figure 9E). These were definitively identified by immunogold labelling with an anti-CdsF antibody, raised against the needle subunit of the chlamydial T3SS (30) (Figures 9F and Figure 10B). The arrays are captured in contact with four membranes *in situ*, forming pathogen synapses at rER-inclusion contact sites with luminal RBs (Figure 11, Movies S6–S8). Strikingly, quantification revealed arrays of T3SS complexes are only observed when RBs contact an inclusion membrane connected to the rER (Figure 11A,B), but not in the absence of ER-inclusion interaction (Figure 11C), or when a smooth membrane contacts the inclusion (Figure 11D). These data reveal a previously unrecognized relationship between the chlamydial T3SS, array assembly and the host rER.

## Discussion

Our data reveal a novel and extensive interaction between mature chlamydial inclusions and the rER that is critical for bacterial infectivity. We show that following an early, bacterial-independent association between calreticulin-containing membranes and *Chlamydia*, mid-cycle inclusions actively and specifically recruit membranes containing transmembrane, luminal and structural components of the host rER. Electron tomography of vitrified freeze-substituted sections of infected cells revealed direct contact and extensive apposition of ribosome-studded rER tubules and the cytoplasmic face of the inclusion membrane. These ER contact sites might potentially represent portals for the selective influx of cellular material into the inclusion, including CERT-dependent ceramide transport (13,14). They can also be specialized sites for bacterial effector translocation, as we show that cytoplasmic rER contacts are exclusively present at sites where the RBs containing assembled T3SSs are simultaneously in contact with the luminal face of the inclusion membrane.

*Legionella* and *Brucella* species also hijack the host ER during the biogenesis of their respective replicative phagosomes. *Legionella*-containing vacuoles become coated in rER membranes that are indirectly recruited through effector-mediated reversible stimulation of host GTPases, pathways sensitive to BFA and NZ (25). The biphasic recruitment of the rER observed during the chlamydial infection cycle is reminiscent of ER recruitment by *Brucella* phagosomes in macrophages, which requires the function of the virulence-associated type IV secretion system (31,32). *Chlamydiae* also apparently manipulate host pathways to uptake host lipids into the inclusion (13,14). This is consistent with our observation of smooth host membranes also in contact with inclusions and invaginations at separate sites, and our confocal imaging showing that endogenous CERT is predominantly partitioned from calreticulin present in the inclusion membrane and lumen. In contrast, we show that chlamydial recruitment of the rER apparently occurs independently of host ER–Golgi and NZ-sensitive transport mechanisms. While interplay between rER recruitment and the previously characterized redirection of secretory trafficking cannot be excluded, our data favour direct recruitment of the rER that may involve as yet unknown bacterial effectors.

The precise role of the host ER in the infection cycle of intracellular bacterial pathogens remains largely unknown (25). In the *Chlamydia* cycle, we show that the rER is not only recruited, but that it is essential for inclusion biogenesis, integrity and bacterial infectivity as indicated by aerolysin sensitivity. During treatment of *Chlamydia*-infected cells, two distinct phenotypes are evident. Early toxin addition prior to rER recruitment arrests the growth of the inclusion, whereas addition following recruitment results in bursting of the inclusion. This implies that interaction with the rER is critical for early inclusion biogenesis, whereas at later stages the inclusion itself has sufficient ER-like character to render it directly susceptible to aerolysin. The latter data are confirmed by labelling of late-stage inclusions with an ER-specific live probe, with similar efficacy to the endogenous organelle. This is also consistent with the presence of phosphatidylinositol-4-phosphate at the inclusion (33). Since ER markers also enter the inclusion and associate with luminal bacteria, ER membranes may be additionally metabolized and potentially incorporated into bacterial envelopes to support growth. Beyond these physical contributions, it remains possible that the functions of the intact rER at the cytosolic face of the inclusion are also central, for instance in the folding or delivery of hydrophobic T3SS effectors, in maintaining the redox state or potential of the inclusion membrane and in the transport of host lipids.

Host inflammatory and immune responses to chlamydial infections are major contributors to the complex disease processes underlying trachoma and infertility. Understanding antigen processing and presentation in *Chlamydia*-infected cells is therefore key, and ER functions are pivotal to many such mechanisms. Indeed, the chlamydial major outer membrane protein, lipopolysaccharide and IncA localize to the ER, where lipopolysaccharide and cellular CD1d colocalize (34). It will now be important to determine the origin and significance of the ER markers observed in close association with bacteria in the inclusion lumen. These accumulate as defined puncta that could represent prior attachment ‘scars’ following dissociation from ER-rich patches of the inclusion membrane or indicate local incorporation of eukaryotic material on the bacterial surface. In either case, it will be critical to examine the fate of this material during the poorly characterized process of cell egress and during the subsequent round of infection.

Our data also define pathogen synapses as novel sites of contact between luminal RBs, the inclusion membrane and the rER. These structures allow the visualization of macromolecular T3SS complexes in contact with four membranes (inner and outer bacterial membranes, inclusion membrane and rER). Although the macromolecular structures observed by Matsumoto in *Chlamydia pscittaci* and isolated inclusions in the absence of intact host cells (5–7) were subsequently proposed to be T3SSs (8), their proportions are inconsistent with the symmetry and dimensions of T3SSs isolated from other bacterial systems (29,35,36). Of course this could reflect a rare variation in this otherwise conserved apparatus (37), or be explained by the relatively destructive preparation methods used at that time. The previously visualized structures have also been proposed to be complexes of the outer membrane protein PmpD (9). We definitively identify the structures at the inclusion membrane as T3SSs by immunogold electron microscopy. In contrast to the earlier structures, their dimensions correlate closely with those of isolated T3SSs. Assembly of the T3SS is apparently associated with dramatic local reorganization of the RB membranes, as seen by the marked widening of the periplasmic space. The arrays containing batteries of T3SSs would ensure the delivery of effectors at high local concentrations. This is the first study where defined bacterial and eukaryotic structures have been imaged together *in situ*. Further biochemical analysis of these synapses will define the bacterial and host components contributing to the formation of these remarkable structures.

Our findings show a key role for the rER in inclusion biogenesis and chlamydial infectivity, through the assembly of the pathogen synapse. Further understanding of these structures will allow new insights into the sophisticated and subversive mechanisms by which pathogenic *Chlamydiae* hijack cellular functions and into the assembly and action of bacterial T3SSs.

## Materials and Methods

### Cells, bacterial strains and reagents

Cell lines (HeLa and RL-95.2) were routinely cultured as recommended by American Type Culture Collection, in 75 cm^2^ tissue culture flasks, 24-well plates or in Lab-Tek (Nunc) chamber slides, as appropriate. *C. trachomatis* serovar LGV2 and D and *C. muridarum* were initially provided by Dr Philippe Verbeke (Institut Jacques Monod, Universite Paris 7-Diderot, France). Bacteria were routinely propagated in HeLa cells as previously described and stored at −80°C in sucrose–phosphate–glucose (SPG) medium for later use (38). Bacterial inclusion-forming units (IFU) were determined as described (38). Culture media (DMEM and HAM's F12), foetal calf serum and gentamycin were purchased from Invitrogen. Brefeldin A (BFA), nocodazole (NZ), chloramphenicol (CHP), ionomycin and valinomycin were from Sigma-Aldrich. Turbofect was from Fermentas. Pro-aerolysin was a generous gift from Professor Gisou van der Goot (Ecole Polytechnique Fédérale de Lausannes, Switzerland). ER blue-white live probe, Mitotracker Orange, WGA-Alexa 594 and Texas Red-conjugated phalloidin were from Invitrogen. Primary antibodies against *Chlamydia* were from Argene, anti-calreticulin, -CERT, -PDI, -RER1, -Derlin-1 were from Sigma, anti-giantin and-HA from Covance and secondary antibodies coupled to different AlexaFluor dyes were from Invitrogen. Goat anti-rabbit secondary coupled to 10 nm gold bead was from British Biocell International. Expression plasmids encoding Rtn4b-HA and DP1-HA were a generous gift from Professor Tom Rapoport (Harvard University, Boston, USA).

### Infection of cultured cells with *Chlamydia*

Cells were seeded at 70% confluence and infected 24 h later by diluting bacterial stock in medium containing gentamycin (infection medium) to give 0.9–1 IFU. Cells were centrifuged (160 × ***g***, 10 min, room temperature) to synchronize the infection, and incubated (80 min, 37°C, 5% CO_2_). The infection medium is then exchanged and cells incubated further until fixation or are prepared for titration.

### Infection of transiently transfected cells with *Chlamydia*

Cells were seeded at 50% confluence and transfected 24 h later using 50 μL of DMEM containing 0.7 μL Turbofect and 150 ng of plasmid DNA per 500 μL of infection medium. The transfection mixture was initially incubated (15 min, room temperature), then mixed with infection medium and subsequently added to cultured cells. Cells are recovered (24 h, 37°C, 5% CO_2_) and infection performed as previously described.

### Labelling of infected cells with Mitotracker Orange

Cultured cells were infected as previously described (38). At the desired point 50 nm of Mitotracker Orange was diluted into the culture medium and cells incubated (45 min, 37°C, 5% CO_2_). Cells were fixed with DMEM containing 4% (w/v) buffered paraformaldehyde (30 min, 37°C, 5% CO_2_). Cells were stained for *Chlamydia* as described.

### Live ER imaging

#### ER-Tracker Blue-White DPX probe

HeLa cells were seeded into Lab-Tek chambers and infected with *C. trachomatis* LGV2 as described; 24 hpi Lab-Tek were transferred onto the microscope stage (Leica DMI 6000), and ER-Tracker Blue-White DPX probe added to the media to a final concentration of 830 nm, and the samples imaged using a filter cube with excitation 340–380 nm, dichroic 400 nm and emission LP 405. An image was captured every 5 seconds for 15 min. Grey levels of inclusions (I), nuclei (N), cytosol (ER) and areas where no cells are present (background) were determined. An intensity ratio was calculated from these values for each time point according to the following formula: (grey level_ER/I_ − mean grey level_background_)/(grey level_N_ − mean grey level_background_). This ratio accounts for photobleaching, variation in probe uptake between cells and intracellular auto-fluorescence. Ratios were determined for each infected cell and the average and standard deviation (shading) calculated.

#### DsRed-ER

HeLa cells were transiently transfected with pcDNA-DsRed-ER, a plasmid encoding DsRed fused to the ER-targeting sequencing of calreticulin at the 3′ end and the ER retention signal (KDEL) at the 5′ end, and infected as previously described; 24 hpi Lab-Tek were transferred onto the microscope stage and inserted into a pre-warmed environmental chamber (Olympus TIRF). A multi-positioning (four positions), z-stack (0.5 µm section depth), time-lapse (every 30 min) experiment was performed for 12 h and images collected using the 60× objective (laser excitation 559 nm, collecting emission 580–700 nm). Data were processed with ImageJ software and a movie frames assembled from maximum intensity projections of confocal stacks at each time point.

### Treatment of infected cells with chemicals and aerolysin

Cells were infected as described previously (38). At appropriate time points (12 or 24 hpi) cells were treated by addition of pro-aerolysin (0.5 nm), valinomycin (2 μm) or ionomycin (2 μm) for 30 min. After 6 h, cells were fixed or samples collected for subsequent titration assays. For drug and antibiotic treatments, agents were added at appropriate time points and concentrations: CHX (12 hpi, 1 or 5 μm), CHP (12 hpi, 5 or 15 μm), NZ (14 hpi, 10 or 20 ng/mL) and BFA (14 hpi, 10 or 20 µg/mL). Cells were fixed at 24 hpi with medium containing 4% (w/v) buffered paraformaldehyde.

### Titration assay

At the time point of interest and after appropriate treatments when necessary, cell layers and supernatants from infected cells were collected in SPG and samples stored at −80°C. HeLa cells were plated at 70% confluence on coverslips. After 24 h, previously collected samples were diluted in infection media and used to infect cells as described. At 24 hpi, cells were fixed as described. Coverslips were immunolabelled using anti-*Chlamydia* antibodies as described, and Hoechst. Coverslips were observed using a fluorescent microscope (Zeiss, Axioskop plus, DAPI cube – Excitation G365/Dichoric FT 395/Emission LP 420, FITC – Zeiss filter set 10 – Excitation BP450–490/ Dichoric FT510/ Emission BP 515–565). Random fields of view were scored to quantify inclusion-forming units per mL (IFU/mL). An average of 400 cells were counted per condition.

### Fluorescence microscopy

For anti-calreticulin, anti-PDI and anti-HA staining, cells were fixed with 4% (w/v) buffered paraformaldehyde in PBS (30 min), and quenched with the same volume of 50 mm NH_4_Cl in PBS. For anti-derlin1, anti-RER1 and anti-giantin staining, cells were fixed with cold methanol (3 min, on ice), before washing with PBS. Cells were permeabilized with methanol/ethanol (5 min, on ice), and non-specific binding blocked by incubation (30 min) with 1% (w/v) BSA in PBS prior to labelling with antibodies diluted appropriately in this blocking buffer. Primary antibodies were incubated (2 h, room temperature or overnight, 4°C), depending on the antibody. Secondary antibodies were incubated (1 h 30 min, room temperature) or for anti-*Chlamydia* conjugated to FITC (1 h, room temperature). When required, Hoechst staining was performed (15 min, room temperature). Alternatively, Texas Red-conjugated phalloidin was used to stain F-actin (30 min, room temperature) after permeabilization, and WGA-Alexa 594 was added prior to permeabilization (15 min, room temperature). Coverslips were mounted on MoWiol.

### Confocal microscopy

Coverslips were observed using a confocal microscope (TCS Sp5 AOBS; Leica). For FITC and Alexa 488, laser at 488 nm was used and emission collected from 498 to 548 nm, for Alexa 546, laser at 543 nm was selected and emission was collected from 555 to 633 nm, for Alexa 594, laser at 594 nm was selected and emission was collected from 605 to 751 nm and for Alexa 633, laser at 633 nm was used and emission collected from 660 to 746 nm. When Hoechst labelling was performed, a Leica spE AOBS inverted microscope was used. Hoechst was excited at 405 nm, and emission was collected from 388 to 517 nm; Alexa 488 was exited at 488 nm and emission was collected from 493 to 619 nm. Each channel was acquired sequentially. Every experiment was performed, acquired and analysed similarly and each duplicate experiment repeated three times.

### Analysis of confocal images

Calreticulin recruitment was analysed using automated quantification in ImageJ (39). Segmentation of the compartment of interest was achieved by applying grey level thresholding on wavelet-transformed images. B-Spline wavelets ‘à trous’ were used for convolution. A mask was created with the ‘Fill Holes’ option selected for the green channel leading to the creation of a volume that represents the inclusion (i.e. the inclusion membrane and the lumen). The proportion of FITC (*Chlamydia*) co-stained with Alexa 568 (calreticulin) or Mitotracker Orange was then determined using the ImageJ Manders coefficient function.

To quantify markers at the inclusion periphery, HeLa cells were infected with *C. trachomatis* LGV2 and fixed 24 hpi prior to triple staining for CERT, calreticulin and *Chlamydia*, as described. Ten inclusions were randomly selected from independent samples and 30 z-views acquired in each channel: CERT (green), calreticulin (red) and *Chlamydia* (blue) spanning the entirety of each inclusion. For every five z-planes (z-depth 0.33 µm; i.e. 6 per inclusion), a single pixel-width trace is made manually around the inclusion edge and the plot profile of each channel saved individually. Grey level values were extracted for each pixel in every channel using Excel and transformed into binary code where 1 > 10 and 0 < 10 (10 was determined to be the background signal in these experiments). The resulting data allow every pixel along each trace to be described as an RGB triplet binary code (e.g. calreticulin+, CERT+, *Chlamydia*− = 110). Pixels representing each subpopulation are summed and expressed as a percentage of the total number of pixels per inclusion (on average 2500 pixels were considered per inclusion). The average and standard deviation are obtained by comparison of populations from independent inclusions.

Three-dimensional (3D) rendering was performed using the 3D viewer plugin in ImageJ [Bibr b39]. When iso-surface was used to generate a model, a threshold of 50 U was used with a transparency of 52% in each channel.

### Transmission electron microscopy

HeLa cells were grown and infected directly on gold-coated copper membrane carriers for HPF with a 100-µm well depth (Leica 707898). At 24 hpi, the membrane carriers containing infected material were transferred to the Leica EM HPM100, where they were rapidly frozen using liquid nitrogen and a pressure of 2100 bar (cooling rate of the sample was approximately 25 000 K/s). The frozen samples were stored in liquid nitrogen until they were freeze-substituted.

Freeze substitution (FS) was performed using the Leica EM AFS2, as described (40). Briefly, membrane carriers were freeze-substituted in dry acetone containing 0.2% uranyl acetate by warming from −160°C to −90°C. The sample was further warmed to −50°C while the FS cocktail was removed and the sample washed in ethanol. The sample was then infiltrated with HM20 resin and the resin polymerized using UV. Finally, the sample was warmed to room temperature and the blocks sectioned in a microtome (Leica UC7).

### Immunogold labelling

Fifty-nanometre sections were incubated for 30 min in blocking buffer [Hanks' balanced salt solution (HBSS) containing 0.2% (v/v) Tween-20, 8% (w/v) BSA, 4.5% (w/v) fish gelatin], followed by incubation with primary antibodies diluted in blocking buffer (1 h, room temperature). Sections were washed once with HBSS containing 0.2% (v/v) Tween-20 and then with HBSS alone, prior to incubation with a goat anti-rabbit secondary conjugated with 10-nm gold beads (45 min, room temperature). Samples were post-fixed with 0.5% (w/v) glutaraldehyde in HBSS and washed extensively.

### Electron tomography

Two hundred-nanometre sections were labelled with protein A coupled to 10 nm gold beads (EMS) as fiducial markers. Dual axis tomograms were collected with SerialEM (41) at a magnification of either 14 000× or 29 000× using a defocus of −1.5 µm on a Tecnai F20 microscope (FEI) equipped with a Gatan 4k CCD camera (Gatan). The tomographic tilt series were collected from −58 to +58 at 2° intervals. Immunolabelled sections were imaged at magnifications ranging from 2000× to 15 000× using Tecnai T10 or T12 microscopes, both equipped with Gatan 1k CCD cameras. Tomograms were aligned and reconstructed using IMOD (42,43). The reconstructions were filtered using nonlinear anisotropic diffusion (44) implemented in IMOD. Tomogram segmentation shown in Figure 5A was performed manually in IMOD.

### Statistical analyses

Data are presented as the mean ± standard deviation of ‘*n*’ experiments, and p-values were calculated using a two-tailed two-sample equal variance Student's *t*-test. A p-value of less than 0.05 was considered statistically significant.

## Supporting Information

**Figure S1: Calreticulin labels the entire *Chlamydia* inclusion.** HeLa cells were infected with *C*. *trachomatis* LGV2 and fixed 24 hpi. Calreticulin (red) and *Chlamydia sp*. (green) were immunolabelled. Images show a typical confocal z-stack acquired sequentially with a z-step of 0.33 μm. Scale bar, 5 μm.

**Figure S2: xz-view of calreticulin recruitment into the *Chlamydia* inclusion.** HeLa cells were infected with *C. trachomatis* LGV2 and fixed 24 hpi. Texas Red-conjugated phalloidin was used to detect F-actin. Calreticulin (red) and *Chlamydia* (green) were immunolabelled. Confocal y-stacks were acquired sequentially with a y-step of 0.5 μm. Left panels show calreticulin in contact with *Chlamydia* in the inclusion. In contrast, phalloidin staining is excluded from the inclusion. This confirms that the calreticulin signal observed in the lumen in the xy-sections of Figures 1 and S1 is not due to deformation of the inclusion. Scale bar, 5 μm.

**Figure S3: ER live probe labels the *Chlamydia* inclusion.** HeLa cells were cultured in Lab-Tek chambers and infected with *C. trachomatis* LGV2. At 24 hpi, chambers containing live infected cells were positioned on the microscope stage, and images acquired every 5 seconds after the addition of the probe to the media. At the end of the acquisition, a differential interference contrast (DIC) image was acquired to confirm the location of the nucleus and inclusions. A) Individual frame from the movie at 470 milliseconds when the probe is incorporated at steady state. Right, selected area from the central image focusing on an infected cell. N, nucleus; I, inclusion. Scale bar, 5 μm. B) Grey level of inclusions (I), nuclei (N), cytosol (ER) and areas where no cells are present (background) were determined. An intensity ratio was calculated from these values for each time point according to the following formula: (grey level_ER/I_ – mean grey level_background_)/(grey level_N_ – mean grey level_background_). This ratio accounts for photobleaching, variation in probe uptake between cells and intracellular auto-fluorescence. Ratios were determined for each infected cell and the average and standard deviation (shading) calculated. As this probe is >520 Da, it will not be taken up passively into the inclusion (10). The graph illustrates that inclusions at 24 hpi are permeable to the ER probe, uptake kinetics of the inclusion are similar to the ER but that inclusions accumulate less probe than the cellular ER.

**Figure S4: 3D rendering of chlamydial inclusions illustrates CERT and calreticulin distribution.** 3D rendering of a representative inclusion formed 24 hpi of HeLa cells with *C. trachomatis* LGV2. Fixed cells were immunolabelled for CERT (green), calreticulin (red) and *Chlamydia* (AlexaFluor 633 pseudocoloured blue). Left and right panels show volume rendering and iso-surface projections of the confocal z-stack, respectively.

**Figure S5: Manual segmentation and model building from tomograms.** HeLa cells were cultured directly on high pressure freezing (HPF) carriers and infected with *C. trachomatis* LGV2. After freeze substitution, Lowicryl HM20 embedded samples were sectioned; 200-nm thick sections were used to record tomograms. Manual segmentation of entire tomograms was performed. An example for tracing is shown (left panel), together with the model superimposed onto a single section (right panel). Modelled structures are colour-coded: eukaryotic ribosomes (red), prokaryotic ribosomes (white), RB outer membrane (purple), RB inner membrane (purple), T3SS (brown), inclusion membrane (bright blue), ER (dark blue), ribosome free membranes (smooth ER, IC, Golgi; green), microtubules (yellow), actin filaments (white). Scale bar, 200 nm.

**Movie S1: Recruitment of DsRed-ER to the *Chlamydia* inclusion in live cells.** HeLa cells transfected with DsRed-ER were infected with *C. trachomatis* LGV2 in Lab-Tek chambers. At 24 hpi, chambers containing live infected cells were transferred onto the microscope stage. Images were acquired every 30 min.

**Movie S2: Uptake of a live ER probe by the *Chlamydia* inclusion.** HeLa cells were infected with *C. trachomatis* LGV2 in Lab-Tek chambers. At 24 hpi, chambers containing live infected cells were transferred onto the microscope stage. After finding the correct focal plane an image (each frame) was acquired every 5 seconds.

**Movie S3: 3D volume rendering of a representative chlamydial inclusion to examine CERT and calreticulin localization.** HeLa cells were fixed 24 hpi after infection with *C. trachomatis* LGV2, then immunostained for CERT (green), calreticulin (red) and *Chlamydia* (AlexaFluor 633 pseudocoloured blue). A confocal z-stack was acquired with a z-dept of 330 nm. Volume rendering was performed using the imagej plugin 3D viewer.

**Movie S4: 3 D iso-surface rendering of a representative chlamydial inclusion to examine CERT and calreticulin localisation.** HeLa cells were fixed 24 hpi after infection with *C. trachomatis* LGV2, then immunostained for CERT (green), calreticulin (red) and *Chlamydia* (AlexaFluor 633 pseudocoloured blue). A confocal z-stack was acquired with a z-depth of 330 nm. Iso-surface rendering was performed using the imagej pugin 3D viewer with a threshold of 50 U for each channel.

**Movie S5: Tomogram and model of the ER interaction with the inclusion membrane and the ‘pathogen synapse’.** The first section steps through the z-stacks used to reconstruct the tomograms presented in 4. This is followed by the model superimposed onto a z-section followed by a rotation to present the three-dimensional volume. Modelled structures are color-coded: eukaryotic ribosomes (red), prokaryotic ribosomes (white), RB outer membrane (purple), RB inner membrane (purple), T3SS (brown), inclusion membrane (bright blue), ER (dark blue), ribosome free membranes (smooth ER, IC, Golgi; green), microtubules (yellow), actin filaments (white).

**Movies S6: Going through the ‘pathogen synapse’.** Video showing stepping through the z-stacks used to assemble the tomograms presented in panels of Figures 9 and 11.

**Movies S7: Going through the ‘pathogen synapse’.** Video showing stepping through the z-stacks used to assemble the tomograms presented in panels of Figures 9 and 11.

**Movies S8: Going through the ‘pathogen synapse’.** Video showing stepping through the z-stacks used to assemble the tomograms presented in panels of Figures 9 and 11.
