# Supplementary material for: Chlamydiae Assemble a Pathogen Synapse to Hijack the Host Endoplasmic Reticulum
Source: Traffic. 2012 Sep 11;13(12):1612–27. doi: 10.1111/tra.12002 (PMC3533787; doi:10.1111/tra.12002)
Supplement: Supplementary file 1 [file tra0013-1612-SD1.doc]

**
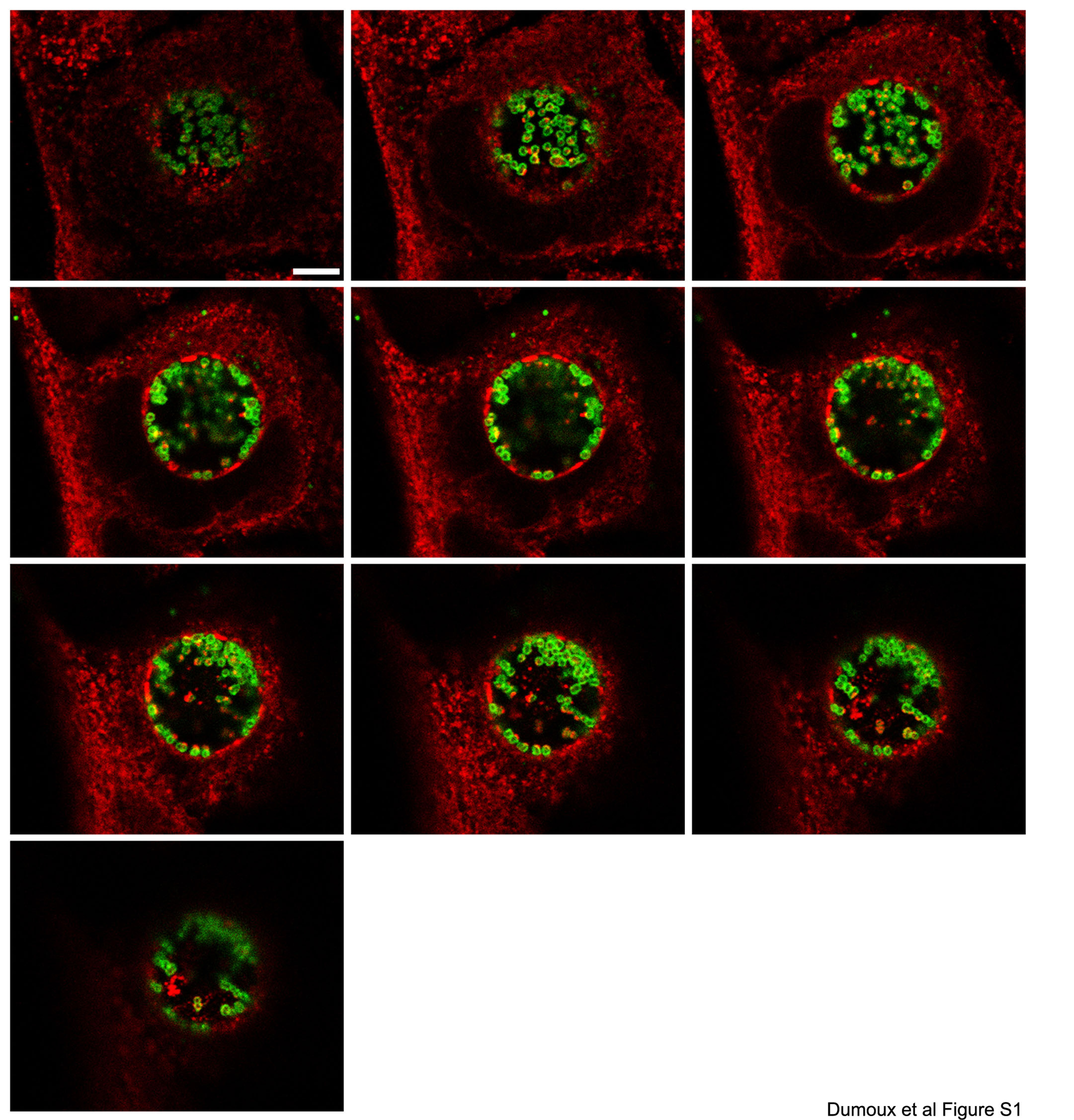
**

**Figure S1: Calreticulin labels the entire *Chlamydia* inclusion.**

HeLa cells were infected with *C.trachomatis* LGV2 and fixed 24 hpi. Calreticulin (red) and *Chlamydia sp.* (green) were immunolabelled. Images show a typical confocal z-stack acquired sequentially with a z step of 0.33 µm. Scale bar, 5 µm.
