# Supplementary material for: Chlamydiae Assemble a Pathogen Synapse to Hijack the Host Endoplasmic Reticulum
Source: Traffic. 2012 Sep 11;13(12):1612–27. doi: 10.1111/tra.12002 (PMC3533787; doi:10.1111/tra.12002)
Supplement: Supplementary file 2 [file tra0013-1612-SD2.doc]

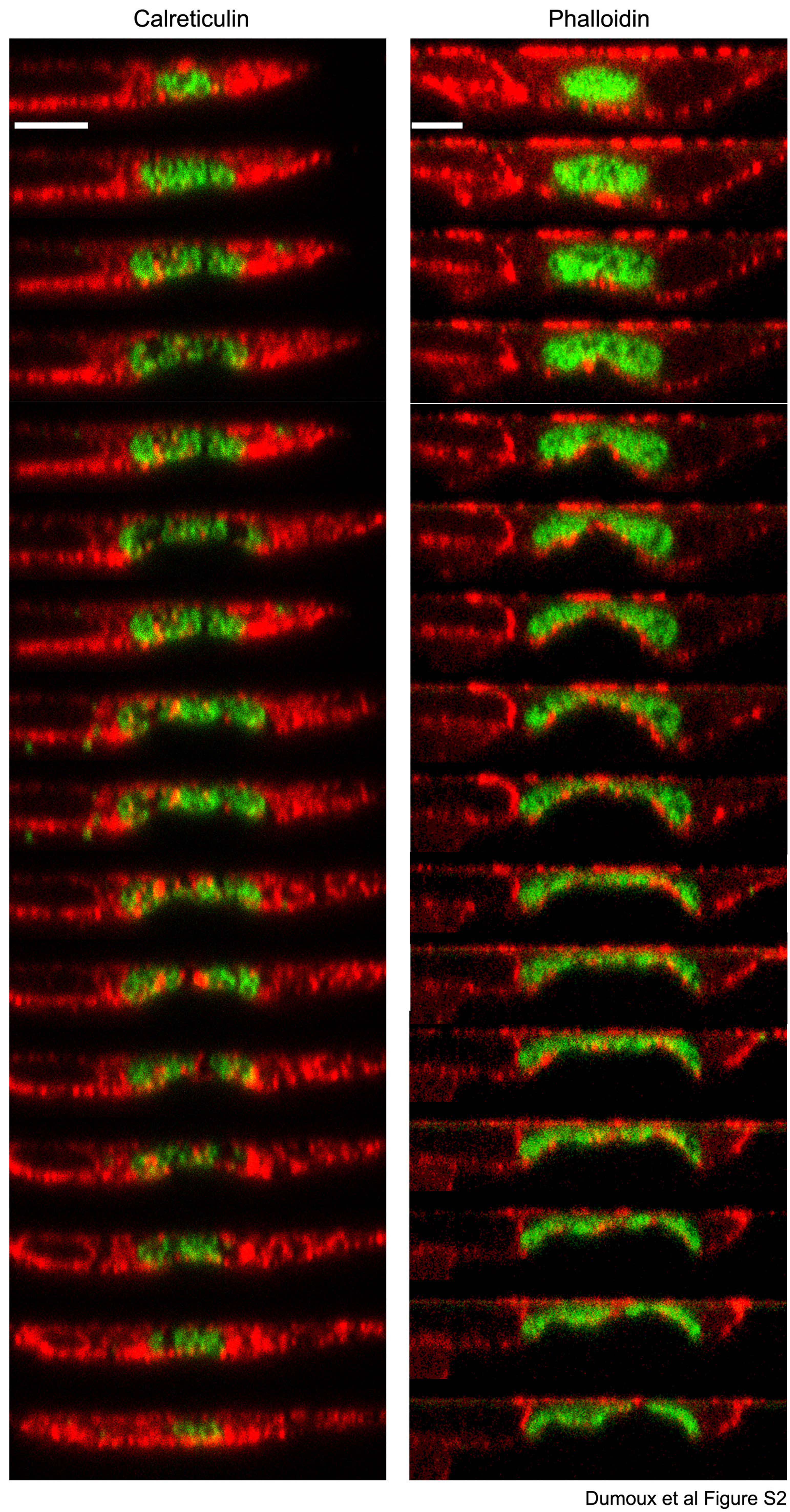


**Figure S2: xz-view of calreticulin recruitment into the *Chlamydia* inclusion.**

HeLa cells were infected with *C.trachomatis* LGV2 and fixed 24 hpi. Texas Red-conjugated phalloidin was used to detect F-actin. Calreticulin (red) and *Chlamydia* (green) were immunolabelled. Confocal y-stacks were acquired sequentially with a y step of 0.5 µm. Left panels show calreticulin in contact with *Chlamydia* in the inclusion. In contrast, phalloidin staining is excluded from the inclusion. This confirms that the calreticulin signal observed in the lumen in the xy-sections of Figures 1 and S1 is not due to deformation of the inclusion. Scale bar, 5 µm.
