# Supplementary material for: Chlamydiae Assemble a Pathogen Synapse to Hijack the Host Endoplasmic Reticulum
Source: Traffic. 2012 Sep 11;13(12):1612–27. doi: 10.1111/tra.12002 (PMC3533787; doi:10.1111/tra.12002)
Supplement: Supplementary file 3 [file tra0013-1612-SD3.doc]

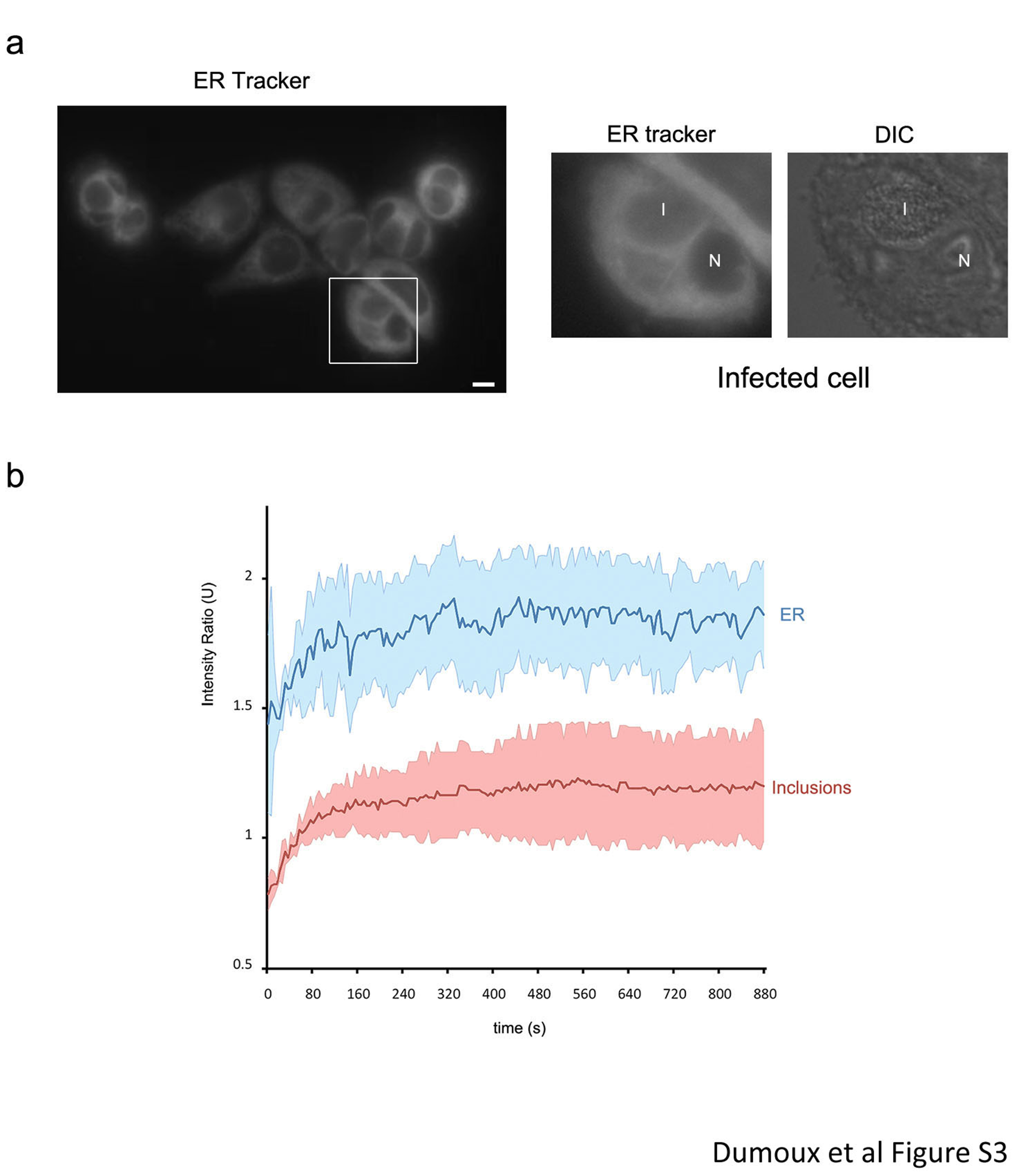


**Figure S3: ER live probe labels the *Chlamydia* inclusion.**

HeLa cells were cultured in Lab-Tek chambers and infected with *C.trachomatis* LGV2. At 24 hpi, chambers containing live infected cells were positioned on the microscope stage, and images acquired every 5 s after the addition of the probe to the media. At the end of the acquisition, a Differential Interference Contrast (DIC) image was acquired to confirm the location of the nucleus and inclusions.

(A) Individual frame from the movie at 470 ms when the probe is incorporated at steady state. Right, selected area from the central image focusing on an infected cell. N: nucleus, I: inclusion. Scale bar, 5 µm.

(B) Grey level of inclusions (I), nuclei (N), cytosol (ER) and areas where no cells are present (background) were determined. An intensity ratio was calculated from these values for each time point according to the following formula: (grey level _ER/I_ – mean grey level _background_)/(grey level _N_-mean grey level _background_). This ratio accounts for photobleaching, variation in probe uptake between cells, and intracellular auto-fluorescence. Ratios were determined for each infected cell and the average and standard deviation (shading) calculated. As this probe >520Da, it will not be taken up passively into the inclusion (10). The graph illustrates that inclusions at 24 hpi are permeable to the ER probe, uptake kinetics of the inclusion are similar to the ER but that inclusions accumulate less probe than the cellular ER.
