# Supplementary material for: Chlamydiae Assemble a Pathogen Synapse to Hijack the Host Endoplasmic Reticulum
Source: Traffic. 2012 Sep 11;13(12):1612–27. doi: 10.1111/tra.12002 (PMC3533787; doi:10.1111/tra.12002)
Supplement: Supplementary file 4 [file tra0013-1612-SD4.doc]

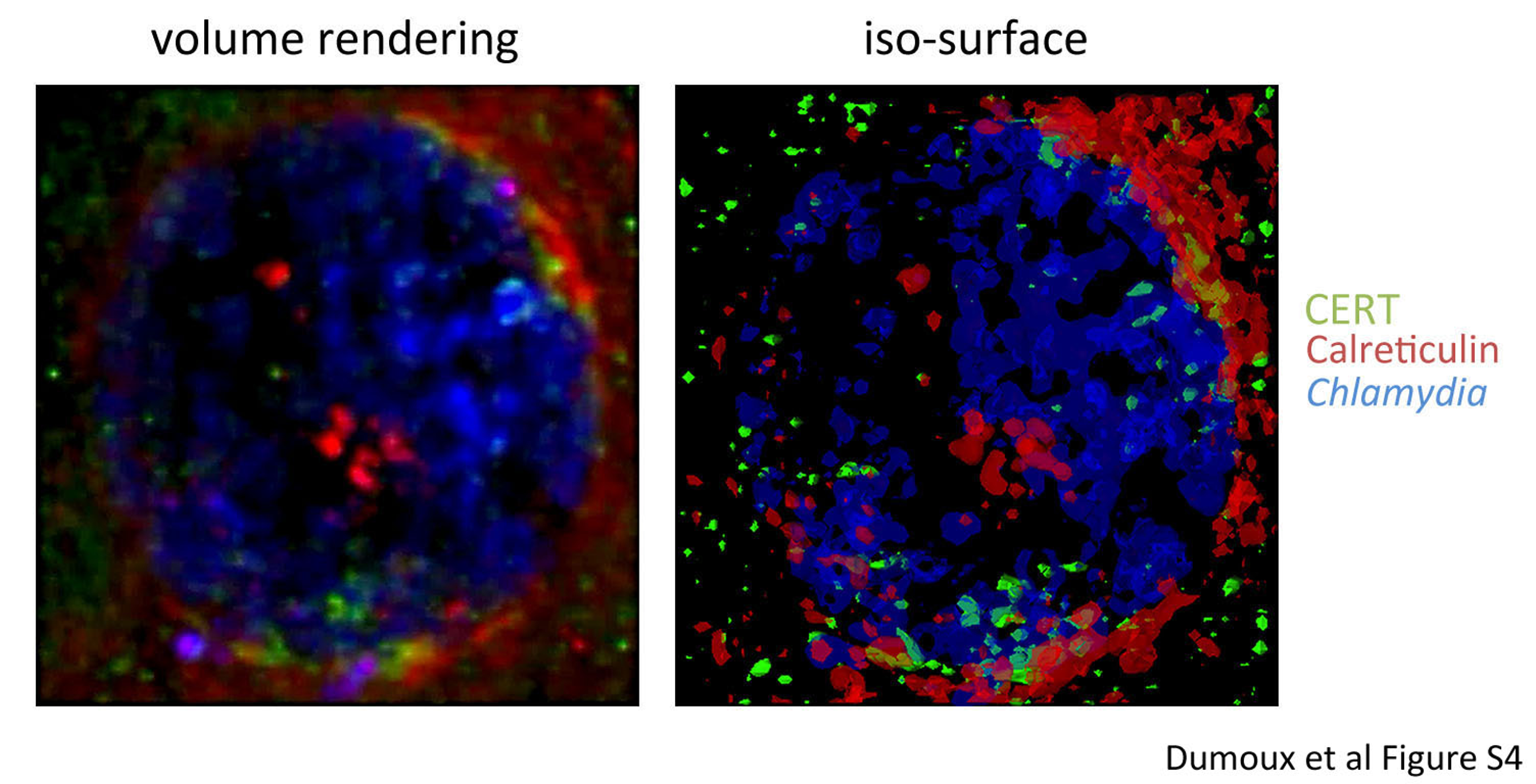


**Figure S4: Three-dimensional rendering of chlamydial inclusions illustrates CERT and calreticulin distribution.**

3D rendering of a representative inclusion formed 24 hpi of HeLa cells with *C.trachomatis* LGV2. Fixed cells were immunolabelled for CERT (green) and calreticulin (red), and *Chlamydia* (AlexaFluor 633 pseudocoloured blue). Left and right panels show volume rendering and iso-surface projections of the confocal z-stack, respectively.
