# Supplementary material for: Chlamydiae Assemble a Pathogen Synapse to Hijack the Host Endoplasmic Reticulum
Source: Traffic. 2012 Sep 11;13(12):1612–27. doi: 10.1111/tra.12002 (PMC3533787; doi:10.1111/tra.12002)
Supplement: Supplementary file 5 [file tra0013-1612-SD5.doc]

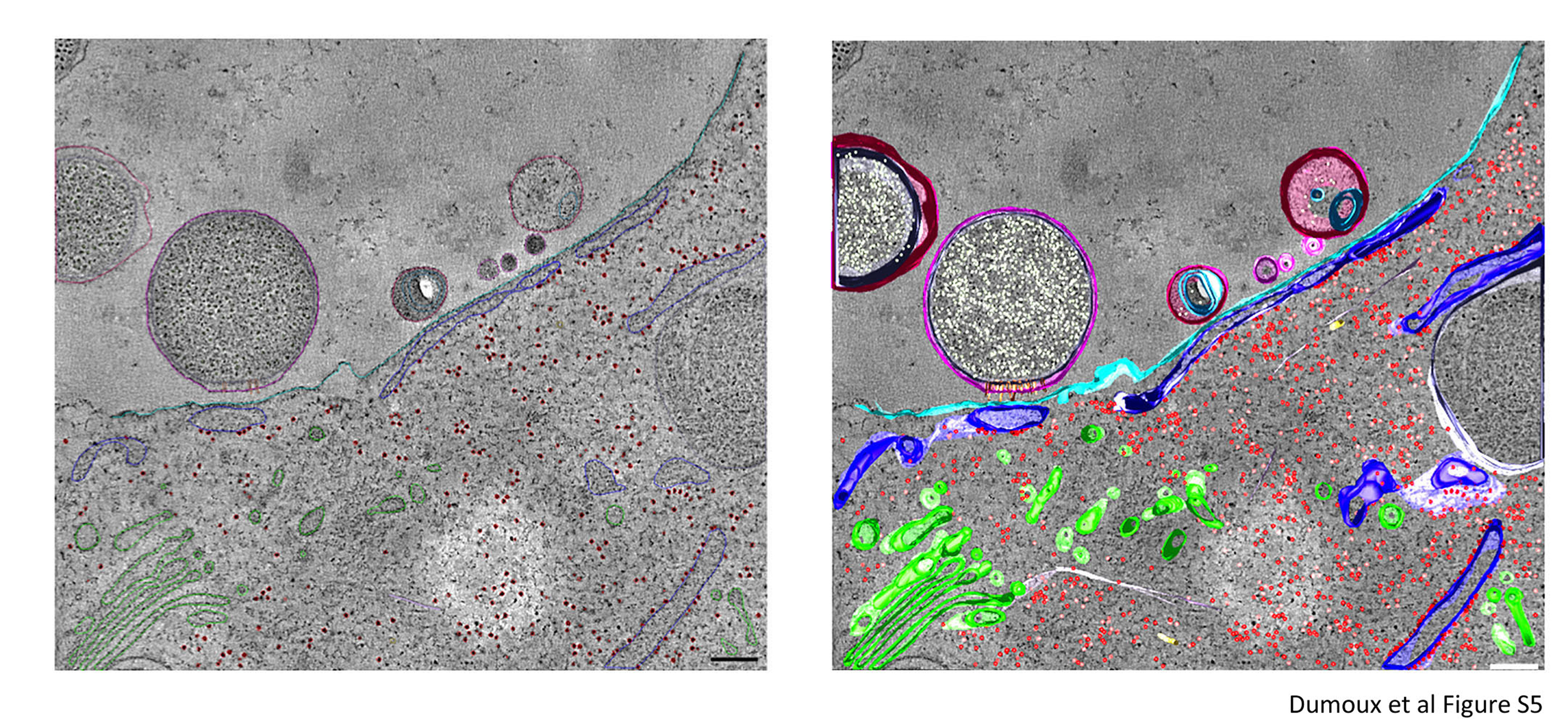


**Figure S5: Manual segmentation and model building from tomograms**

HeLa cells were cultured directly on high pressure freezing (HPF) carriers and infected with *C.trachomatis* LGV2. After freeze-substitution, Lowicryl HM20 embedded samples were sectioned. 200 nm thick sections were used to record tomograms. Manual segmentation of entire tomograms was performed. An example tracing is shown (left panel), together with the model superimposed onto a single section (right panel). Modelled structures are colour-coded: eukaryotic ribosomes (red), prokaryotic ribosomes (white), RB outer membrane (purple), RB inner membrane (purple), type III secretion system (T3SS; brown), inclusion membrane (bright blue), ER (dark blue), ribosome free membranes (smooth ER, IC, Golgi; green), microtubules (yellow), actin filaments (white). Scale bar, 200 nm.
